# Simultaneous LC-MS/MS-Based Quantification of Free 3-Nitro-l-tyrosine, 3-Chloro-l-tyrosine, and 3-Bromo-l-tyrosine in Plasma of Colorectal Cancer Patients during Early Postoperative Period

**DOI:** 10.3390/molecules25215158

**Published:** 2020-11-05

**Authors:** Mariusz G. Fleszar, Paulina Fortuna, Marek Zawadzki, Bogna Kosyk, Małgorzata Krzystek-Korpacka

**Affiliations:** 1 Department of Medical Biochemistry, Wroclaw Medical University, 50-368 Wroclaw, Poland; paulina.fortuna@umed.wroc.pl; 2 Department of Oncological Surgery, Regional Specialist Hospital, 51-124 Wroclaw, Poland; zawadzki@wssk.wroc.pl; 3 Department of Physiotherapy, Wroclaw Medical University, 51-618 Wroclaw, Poland; 4 Institute of Soil Science and Environmental Protection, Wroclaw University of Environmental and Life Sciences, 50-375 Wroclaw, Poland; bogna.kosyk@upwr.edu.pl

**Keywords:** metabolomics, liquid chromatography–tandem mass spectrometry, oxidative stress, nitrosative stress, inflammation, minimal invasive surgery, biomarkers

## Abstract

Quantification with satisfactory specificity and sensitivity of free 3-Nitro-l-tyrosine (3-NT), 3-Chloro-l-tyrosine (3-CT), and 3-Bromo-l-tyrosine (3-BT) in biological samples as potential inflammation, oxidative stress, and cancer biomarkers is analytically challenging. We aimed at developing a liquid chromatography–tandem mass spectrometry (LC-MS/MS)-based method for their simultaneous analysis without an extract purification step by solid-phase extraction. Validation of the developed method yielded the following limits of detection (LOD) and quantification (LOQ) for 3-NT, 3-BT, and 3-CT: 0.030, 0.026, 0.030 ng/mL (LODs) and 0.100, 0.096, 0.098 ng/mL (LOQs). Coefficients of variation for all metabolites and tested concentrations were <10% and accuracy was within 95–105%. Method applicability was tested on colorectal cancer patients during the perioperative period. All metabolites were significantly higher in cancer patients than healthy controls. The 3-NT was significantly lower in advanced cancer and 3-BT showed a similar tendency. Dynamics of 3-BT in the early postoperative period were affected by type of surgery and presence of surgical site infections. In conclusion, a sensitive and specific LC-MS/MS method for simultaneous quantification of free 3-NT, 3-BT, and 3-CT in human plasma has been developed.

## 1. Introduction

The transient overproduction of reactive oxygen (ROS) and nitrogen species (RNS) accompanies an acute inflammatory reaction and enables the rapid elimination of invading pathogens. When sustained, however, it can cause damage to macromolecules and contribute to the initiation and progression of a number of diseases, including cancer [1,2]. Proteins are major targets for oxidative/nitrosative damage by virtue of being the most abundant biomolecules [3]. Aromatic tyrosine residues in proteins are particularly susceptible to modification by ROS and RNS and, unlike for sulfur-containing amino acids, the change is considered irreversible [4]. Due to its key role in signal transduction pathways, damage to tyrosine may have a greater impact than modification of any other amino acid.

The 3-Nitro-l-tyrosine (3-NT) is the most common derivative, resulting from tyrosine nitration by the products of nitric oxide (NO), mainly peroxynitrite or nitrogen dioxide. Nitric oxide synthases (NOS) are the key NO source. Inflammation- and hypoxia-induced enzyme isoform (iNOS/NOS2) produces NO at concentrations several orders of magnitude higher than constitutive NOS1 and NOS3 [5] and therefore is mainly responsible for the synthesis of 3-NT. The macrophage NOS2 has been implicated in cancer promotion [6,7]. Local NOS2 overexpression has been shown in the digestive tract tumors [8,9], including those in the colon and rectum [10], predicting poor prognosis [11].

Oxidative burst accompanies the activation of phagocytes during stress responses. Myeloperoxidase (MPO), expressed by neutrophils and monocytes, preferentially uses chloride to form hypochloric acid, leading to the formation of 3-Chloro-l-tyrosine (3-CT). The MPO has been implicated in the initiation of neoplastic transformation in animal models of colitis-associated cancer [12] and the enzyme expression indicates an increased risk for colorectal cancer (CRC) in humans [13]. In turn, eosinophil peroxidase (EPO) preferentially uses bromide to form hypobromic acid with 3-Bromo-l-tyrosine (3-BT) as a by-product [14,15]. Interestingly, activated eosinophils have been shown to play an antitumorigenic role in CRC [16].

Tyrosine modified by ROS and RNS can serve as a stable oxidative stress marker, a prognostic marker in cancer [17], and as an indicator of NOS2, MPO, or EPO activity [15] and therefore a marker of inflammatory leukocyte-mediated tissue damage [18]. The concomitant quantification of 3-NT, 3-CT, and 3-BT may enhance the diagnostic and prognostic power of individual determinations and provide a unique insight into the role of tyrosine modifications in cancer. However, currently available methods lack sensitivity [19] and/or are dedicated to the quantification of pairs: 3-NT and 3-CT [20,21] or 3-NT and 3-BT [22,23]. Gaut et al. [19] developed a highly sensitive gas chromatography–mass spectrometry (GC-MS)-based method for the concomitant evaluation of all tyrosine derivatives but the liquid chromatography–tandem mass spectrometry (LC-MS/MS)-based approach did not allow for the analysis of biological samples. Here, we reduced the number of purification steps and used a different extraction solvent, which allowed us to obtain an efficient and sensitive LC-MS/MS-based method for the simultaneous analysis of 3-NT, 3-CT, and 3-BT in human plasma. The developed method was applied to determine the association between products of tyrosine modifications and CRC presence and advancement as well as their dynamic in the early postoperative period and association with adverse clinical outcomes following surgery.

## 2. Results

### 2.1. Method Development

Due to the polarity of quantified metabolites and their theoretical pKa values (close to 2), the first step was to select an appropriate extraction reagent. Acetonitrile, methanol, acetone, and ethyl acetate were selected as potential extraction reagents. Of these, ethyl acetate was tested because of the relatively high solubility of unmodified tyrosine in this particular solvent [24] as well as the comparatively high octanol–water partition coefficient and low boiling point. The above-mentioned features are potentially advantageous as they facilitate extract evaporation during the necessary concentration step in the vacuum evaporator. Unfortunately, the recoveries obtained with the extraction protocol utilizing ethyl acetate were low, which necessitated the use of an extraction reagent with higher relative polarity. This resulted in a longer concentration time but allowed us to improve sensitivity. The second step was to select a stabilization buffer used to optimize the samples’ pH, which would allow for obtaining a sufficiently high recovery rate. Theoretical pKa values indicate that 3-NT, 3-CT, and 3-BT are moderately acidic compounds. Therefore, lowering the pH should result in non-ionized forms of quantified metabolites, which, in turn, would translate into higher recovery rates [25]. Indeed, lowering the pH resulted in a significant improvement of the extraction coefficient. Initially, formic acid (FA, 0.1% and 0.2% in water), trifluoroacetic acid (TFA, 0.1% and 0.2% in water), and sulfuric acid (1.8 M) solutions were tested. Particular attention has to be paid to the stability of the standard solutions. We have tested stock solutions of standards, with concentrations below 100 and above 100 µg/mL stored at 4 °C and previously prepared calibration standards frozen at −20 °C. Peaks’ responses of calibration points obtained from the respective solutions were measured for 3 months at 4-day intervals. Response coefficients of variation (CVs) below 15% were considered as acceptable. Our tests showed that only stock solutions above 100 µg/mL are stable at 4 °C for 2 months. All calibration standards should be prepared freshly before analysis, for which large volumes of blank plasma are needed or, if unavailable, the blank plasma can be replaced with an artificial matrix. Acetone was used as an optimal extraction and deproteinization reagent and 0.2% TFA as an acidifying buffer.

Here, the calibration curves were made by dissolving the appropriate standard stock solutions in water. To minimize matrix effects for all tested metabolites, stable, isotopically labeled internal standards (3-Nitro-l-tyrosine (RING-13C6), 3-Chloro-l-tyrosine (RING-13C6) and 3-Bromo-l-tyrosine (RING-13C6)) were used. In order to verify the method’s suitability for plasma measurements, the analysis of the method’s accuracy, precision, and recovery was performed by the addition of working standard solutions to plasma samples. Following the European Medicines Agency (EMA) guidelines [26,27], the developed method was validated and its parameters, such as linearity, accuracy, precision, and recovery, as well as limits of detection and quantification, were determined.

### 2.2. Method Validation

#### 2.2.1. Linearity

Calibration curves were prepared by dissolving stock solutions in water to yield the concentrations of 0.1, 0.2, 0.5, 1.0, 1.5, 2.0, 2.5, and 3.0 ng/mL. Calibration curve linearity was determined by calculating the coefficient of determination (acceptable threshold: ≥0.995) and by conducting a *t*-test for slope significance. The working range of the calibration curve was determined by the assessment of homogeneity of variances of the lowest and highest calibration levels using the Snedecor *F* test. The calculated *F* value should be equal to or below the expected *F* value. The following formula was applied to the established *F* value: *F*cal = the highest relative standard deviation (RSD) for calibration level/the lowest RSD for calibration level.

#### 2.2.2. Accuracy and Precision

Spiked plasma samples at low, medium, and high concentrations of 0.275, 1.5, 2.5 ng/mL were used to determine the accuracy and precision. The coefficient of variation (CV) for each level should be below 15%, which is consistent with EMA guidelines for the validation of bioanalytical methods [26,27].

#### 2.2.3. Recovery

Recovery rates were evaluated at a concentration of 0.3, 0.6, 1.3 ng/mL, prepared by adding 2 µL of proper working standard solution to 98 µL of plasma. The following equation was applied: [(response obtained for spiked plasma – response of non-spiked plasma)/response of working standard solution diluted in water × 100]. Recovery rates should be in the range of 80–120%.

#### 2.2.4. Limit of Detection and Limit of Quantification

The limit of detection (LOD) and the limit of quantification (LOQ) were determined based on standard deviation (SD) of the response and the slope of calibration curves. The SD values of the response were estimated as standard deviation of y-intercepts of regression curves. The following formulas were applied: [3.3× SD/slope] for LOD and [10× SD/slope] for LOQ.

The calculated *F* values were 4.25, 3.19, 1.60 for 3-NT, 3-CT, 3-BT, respectively. They were obtained for *n* = 6 and *α* = 0.05 and were below the expected *F* value (*F*_crit_ = 5.05), indicative of the correctness of the calibration curve range.

**Figure 1:**
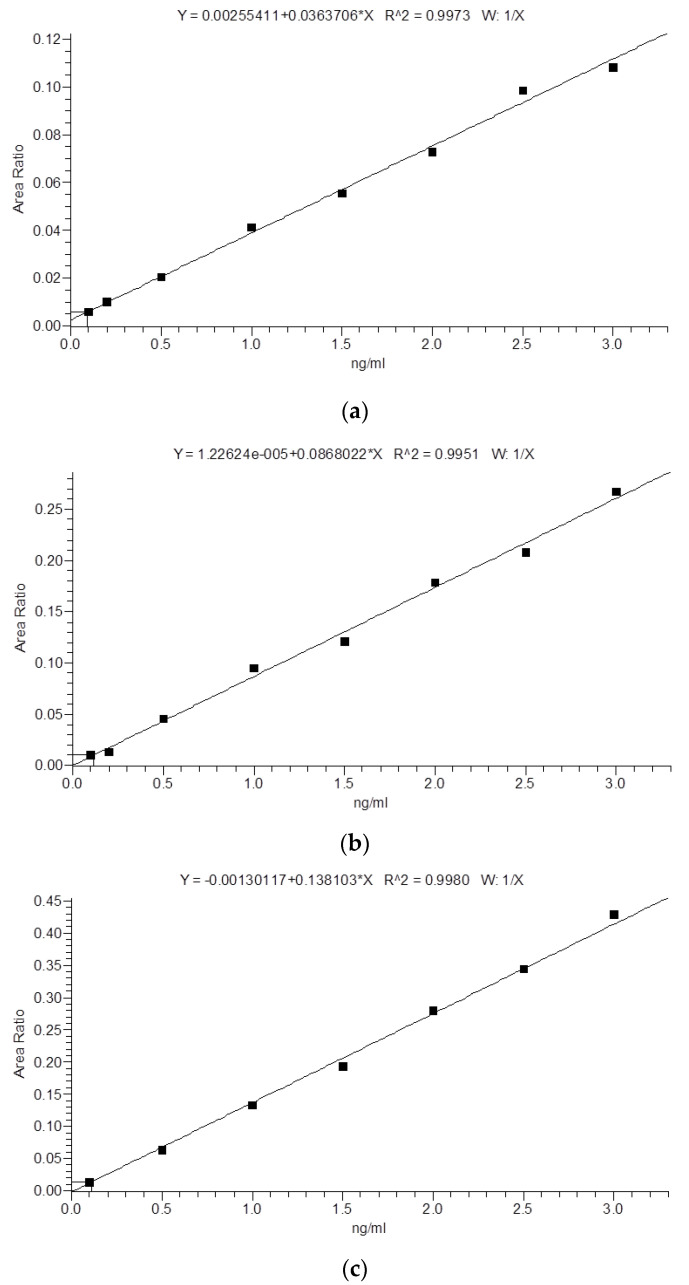
Calibration curve for the LC-MS/MS analysis of (**a**) 3-Nitro-l-tyrosine (3-NT); (**b**) 3-Chloro-l-tyrosine (3-CT); (**c**) 3-Bromo-l-tyrosine (3-BT).

**Figure 2:**
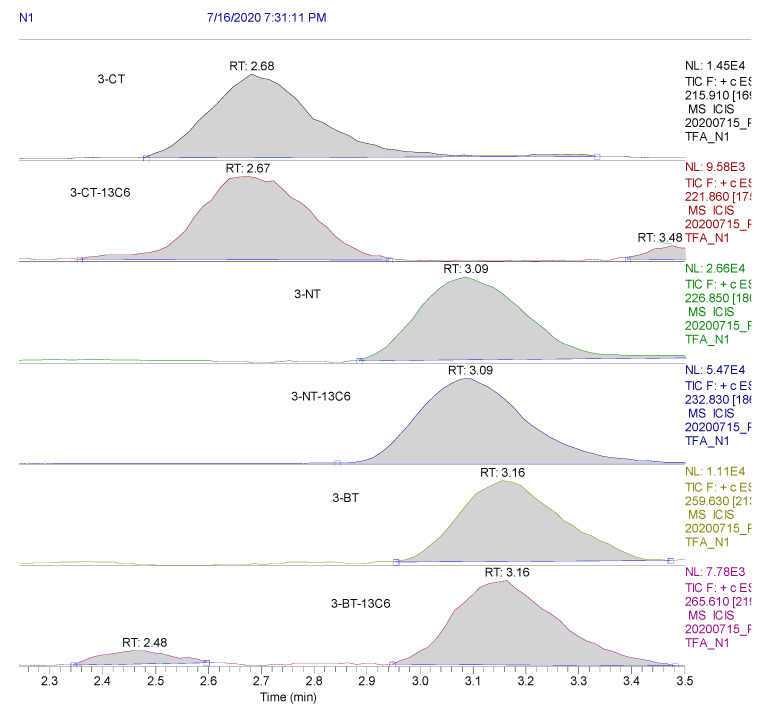
Ion chromatograms of 3-CT, 3-CT-13C6 (3-Chloro-l-tyrosine (RING-13C6)), 3-NT, 3-NT-13C6 (3-Nitro-l-tyrosine (RING-13C6)), 3-BT, and 3-BT-13C6 (3-Bromo-l-tyrosine (RING-13C6)).

Moreover, all other tested validation parameters met the assumed criteria. The obtained results are presented in Table 1 and Table 2. Calibration curves and exemplary ion chromatograms are shown in Figure 1 and Figure 2.

### 2.3. Application of the Method to Determine Plasma Concentrations of Oxidatively Modified Tyrosine in CRC Patients

The applicability of the devised method was tested in a clinical setting in colorectal cancer patients at baseline and in a 72-h follow-up after curative tumor resection.

#### 2.3.1. Plasma 3-NT, 3-CT, and 3-BT in CRC Patients and Healthy Individuals

**Figure 3:**
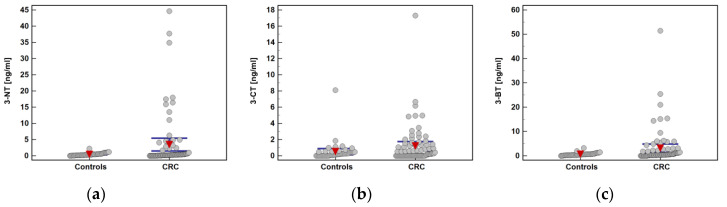
Individual metabolite concentrations in study population: (**a**) 3-Nitro-l-tyrosine (3-NT); (**b**) 3-Chloro-l-tyrosine (3-CT); (**c**) 3-Bromo-l-tyrosine (3-BT). Red triangles with whiskers represent mean with 95% confidence interval. CRC, colorectal cancer.

Plasma concentrations of 3-NT, 3-CT, and 3-BT were analyzed in 75 CRC patients and 43 apparently healthy controls. Both groups were well matched with respect to age and sex distribution (Table 3). Comparative analysis showed that 95.3% of controls and 85.3% of CRC patients had quantifiable concentrations of 3-NT (*p* = 0.129). The 3-BT was quantifiable in 93% of controls and 88% of CRC patients (*p* = 0.532) and 3-CT in 74.4% of controls and 60% of CRC patients (*p* = 0.159). Mean concentrations of all analytes were significantly lower in healthy controls than CRC patients (Table 3). The distribution of individual metabolite concentrations is presented in Figure 3.

##### Effect of Patient-Related Features on Plasma 3-NT, 3-CT, and 3-BT

Sex or age had no effect on 3-NT (*p* = 0.524 and *p* = 0.647), 3-CT (*p* = 0.228 and *p* = 0.989), or 3-BT (*p* = 0.445 and *p* = 0.855) in CRC patients. Moreover, BMI or patient’s physical status expressed as the American Society of Anesthesiologists (ASA) score or the Charlson comorbidity index (CCI) had no effect on 3-NT (respectively, *p* = 0.138, *p* = 0.935, and *p* = 0.757), 3-CT (respectively, *p* = 0.747, *p* = 0.620, and *p* = 0.252), and 3-BT (respectively, *p* = 0.612, *p* = 0.787, and *p* = 0.662).

##### Effect of Cancer-Related Features on Plasma 3-NT, 3-CT, and 3-BT

Of the cancer-related features, 3-NT was significantly lower in patients with cancers metastasizing to lymph nodes (0.97 ng/mL (0.42–1.51) in N(+) vs. 4.48 ng/mL (1.23–7.74) in N0, *p* = 0.037) and 3-BT displayed a similar tendency (1.62 ng/mL (0.59–2.65) in N(+) vs. 4.44 ng/mL (1.28–7.59), *p* = 0.092), while 3-CT was not associated with lymph node metastasis (*p* = 0.645). The 3-NT negatively correlated with tumor grade (ρ = −0.29, *p* = 0.024) and 3-BT displayed a similar tendency (ρ = −0.25, *p* = 0.055). In addition, 3-NT was significantly lower in patients with distant metastases (0.83 ng/mL (0–2.15) in M1 vs. 2.99 ng/mL (1.1–4.89) in M0, *p* = 0.047) while 3-CT displayed the opposite trend (2.13 ng/mL (0–4.51) in M1 vs. 0.87 ng/mL (0.52–1.23) in M0, *p* = 0.073).

##### Correlation Pattern of Plasma 3-NT, 3-CT, and 3-BT

In CRC patients, 3-NT was correlated with 3-CT (ρ = 0.40, *p* < 0.001) and 3-BT (ρ = 0.27, *p* = 0.020), while in controls, only 3-CT and 3-BT concentrations were correlated (ρ = 0.38, *p* = 0.012). Of other inflammatory mediators, 3-NT in CRC patients correlated positively with PDGF-BB (ρ = 0.24, *p* = 0.043) and MIP-1β (ρ = 0.24, *p* = 0.044).

#### 2.3.2. Plasma 3-NT, 3-CT, and 3-BT in CRC Patients during Early Perioperative Period

**Figure 4:**
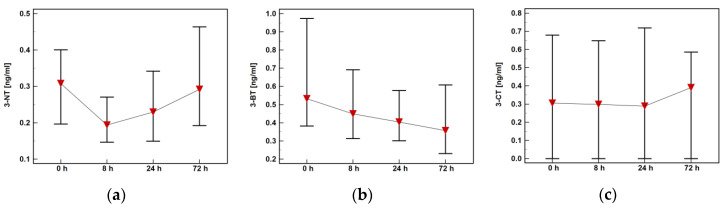
Plasma concentrations of oxidatively modified tyrosine in colorectal cancer patients during early postoperative period: (**a**) 3-Nitro-l-tyrosine (3-NT); (**b**) 3-Chloro-l-tyrosine (3-CT); (**c**) 3-Bromo-l-tyrosine (3-BT).

All metabolites displayed distinct postoperative dynamics. The 3-NT dropped at 8 h post-incision but was rising afterwards, while 3-BT was steadily decreasing. The 3-CT was rather stable during follow-up but metabolite concentration showed the highest variability (Figure 4).

**Figure 5:**
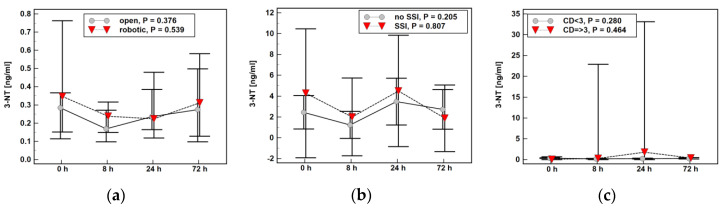
Impact of surgery-related factors on a time course of 3-Nitro-l-tyrosine (3-NT): (**a**) type of surgery; (**b**) surgical site infections (SSI); (**c**) the Clavien–Dindo (CD) classification of surgical complications.

**Figure 6:**
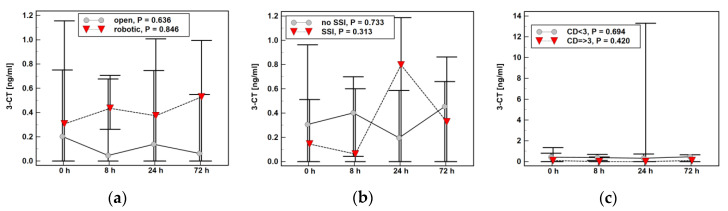
Impact of surgery-related factors on a time course of 3-Chloro-l-tyrosine (3-CT): (**a**) type of surgery; (**b**) surgical site infections (SSI); (**c**) the Clavien–Dindo (CD) classification of surgical complications.

The time profile of 3-NT (Figure 5) or 3-CT (Figure 6) was not significantly affected by type of surgery (open vs. robotic), presence of surgical site infections (SSI), or serious surgical complications (expressed as score ≥3 in the Clavien–Dindo classification).

**Figure 7:**
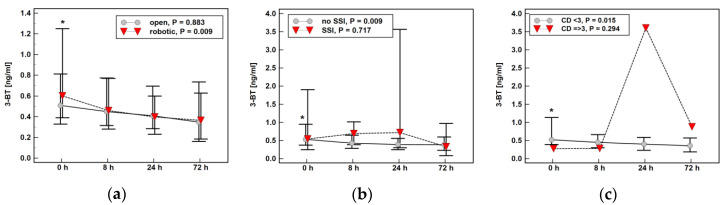
Impact of surgery-related factors on a time course of 3-Bromo-l-tyrosine (3-BT): (**a**) type of surgery; (**b**) surgical site infections (SSI); (**c**) the Clavien–Dindo (CD) classification of surgical complications. *, significantly different from concentrations at other time points.

The time profile of 3-BT concentration over time was affected by surgery type—patients undergoing robotic surgery experienced a more marked drop at 8 h post-incision than those undergoing open procedure (Figure 7a). A steady decrease in 3-BT was also observed in patients without SSI (Figure 7b) or serious surgical complications (Figure 7c) as opposed to a metabolite increase in patients with SSI or serious surgical complications (mostly anastomotic leak).

## 3. Discussion

There is growing interest in the quantification of 3-Nitro-l-tyrosine (3-NT), 3-Bromo-l-tyrosine (3-BT), and 3-Chloro-l-tyrosine (3-CT) as markers of inflammation and oxidative and nitrosative stress. The available methods are based on gas chromatography–mass spectrometry (GC-MS) [19,28,29,30] or LC-MS [18,22,23,31,32,33] analysis or on affinity approaches (immunoassays) [34,35]. Owing to the relatively high polarity of 3-NT, 3-CT, and 3-BT, and in order to improve their thermal stability, the GC-MS-based methods require prior sample derivatization [19,28,29]. For a non-derivative analysis, either LC-MS or affinity approaches can be used. However, both techniques have their limitations as well. Most LC-MS methods have an additional step of purifying obtained extracts using a solid-phase extraction (SPE) technique [20,22,31,36], increasing costs, and time required for sample preparation. Regarding immunoassays, the biggest concern is their insufficient selectivity and specificity due to cross-reactivity with tyrosine and other structurally similar metabolites [37,38], but the immunoassay sensitivity is not optimal either [38]. These shortcomings make it difficult to compare the immunoassay results obtained in different studies [38]. Consequently, more sensitive, selective, and specific MS-based techniques, especially those allowing for analysis in the MS/MS mode, are considered to be methods of choice [37]. There are two main approaches for 3-NT, 3-CT, and 3-BT quantification: in a protein-bound form or in a free, circulating form. Owing to low concentrations of free forms, quantification of protein-bound modified tyrosines is a more popular approach. However, it has also a greater potential for artifacts. With the analysis of protein-bound forms, particular attention should be paid to the choice of hydrolysis method. Although chemical hydrolysis has superior efficiency over enzymatic hydrolysis, it is more prone to the formation of artifacts [37,38]. Here, we propose an efficient and sensitive method for the simultaneous analysis of circulating 3-NT, 3-CT, and 3-BT in human plasma using an LC-MS/MS technique utilizing a liquid–liquid extraction method without extracts needing SPE cleaning and derivatization steps.

Most of the quantitative methods focus on 3-NT [28,29,31,32] and some determine the pairs of 3-NT and 3-CT [20,21] or 3-NT and 3-BT [22,23]. To the best of our knowledge, there is only one method utilizing the LC-MS/MS technique, in which an attempt at concomitant 3-NT, 3-CT, and 3-BT determination has been made. However, the sensitivity of the developed method has not allowed for quantitative analysis of the metabolites [19]. The authors have compared their results with the data obtained by GC-MS, which they found to be superior as the quantification limits were 0.07 fM, 0.005 fM, 0.19 fM for 3-NT, 3-CT, and 3-BT, respectively. However, the GC technique applied there requires prior sample derivatization and the use of a non-standard electron capture–negative chemical ionization technique [19]. A comparative analysis of GC-MS and LC-MS conducted by Yi et al. [32] showed that the high temperature and low pH needed to carry out the derivatization with trifluoroacetic anhydride as derivatization reagent can lead to the generation of artifactual 3-NT in biological samples [32]. Additionally, the LOD for 3-NT obtained with their method (0.5 ng/mL) is significantly higher than the one reported here (0.03 ng/mL). Apart from being at increased risk of artifacts associated with the derivatization step, another method utilizing a GC-MS technique demonstrated by Schwedhelm et al. [39] yielded almost three times higher LOQ than the one obtained with our method (125 pM against 44.2 pM). In turn, Hui et al. [31] utilized the LC-MS technique for the quantification of free 3-NT but obtained unsatisfactory LOQ (0.625 nM). The probable reason is the use of O-methyl-l-tyrosine as an internal standard instead of an isotope-labeled derivative [31]. An interesting approach has been undertaken by Song et al. [21]. It was based on a direct analysis in real time using tandem mass spectrometry, allowing for extremely simple and quick preparation of samples. Unfortunately, the resulting LOQs of 0.6 µg/mL for 3-NT and 0.3 µg/mL for 3-CT were far above the concentrations recorded in human plasma [21]. In conclusion, the method developed here is one of the few dedicated to the analysis of circulating forms of 3-NT, 3-CT, and 3-BT in human plasma [21,29,31,32,39] and the only one that provides the possibility of simultaneous quantification all three of them.

We tested the potential applicability of the developed method on a cohort of CRC patients whose plasma samples were collected at baseline and in a short postoperative follow-up. A similar percentage of analyzed samples had unmeasurable concentrations of 3-NT and 3-BT—up to 7% in controls and 15% among CRC patients. In turn, 3-CT concentrations were significantly more frequently below the limit of detection than 3-NT and 3-BT in both cohorts. Regarding comparison of CRC patients and healthy individuals, no significant differences in the percentage of individuals with 3-NT, 3-CT, and 3-BT levels below the method’s limit of detection were observed, although 3-NT and 3-CT tended to be more frequently unquantifiable in the CRC group. Nonetheless, the concentrations of all evaluated modified tyrosines were significantly elevated in CRC patients as compared to apparently healthy controls. Still, 3-NT significantly and 3-BT insignificantly were lower in patients with advanced disease in terms of depth of tumor invasion and local and distant metastases. However, regarding distant metastases, this observation needs to be interpreted with care as only a limited number of patients had metastases in distant organs. Nonetheless, concerning 3-BT, this observation is consistent with the anti-tumorigenic role attributed to eosinophils in CRC [16,40]. It also corroborates findings of an inverse relationship between tumor infiltration by eosinophils and the metastatic potential of colorectal cancer as well as the progression from adenoma to carcinoma (reviewed in [41]). The 3-NT is considered a footprint of NOS2 activity [5], and NOS2 expression in tumors of the digestive tract is elevated [8,9,10]. Therefore, the inverse 3-NT correlation with lymph node and distant metastasis observed here seems counterintuitive. However, Teng et al. [42] attributed an inhibitory role to 3-NT in angiogenesis, as the metabolite uncoupled endothelial nitric oxide synthase (eNOS) and promoted the generation of superoxide anion over NO. Accordingly, others have shown 3-NT to impair the function of vascular endothelial cells, inhibit the proliferation of vascular smooth muscle cells, and remodel the cytoskeleton [43,44,45,46]. Moreover, while the oxidative stress is clearly pro-tumorigenic in early stages of neoplastic transformation, the excessive production of oxygen and nitrogen radicals at later stages may become disadvantageous, causing destabilization of the tumor cell genome and promoting cell senescence and death [2,47]. Furthermore, the immunosuppressive environment and its key mediators, including IL-4 and IL-13, elevated in CRC [48], inhibit NOS2 expression in macrophages [49]. Interestingly, only the concentration of 3-CT tended to increase with CRC advancement. The 3-CT is considered an in vivo marker of MPO activity [50]. The level of local expression of MPO in the bowel directly translates into oxidative stress at the systemic level, even in healthy individuals [51]. Excessive MPO activity is involved in the pathogenesis of inflammatory bowel disease and in the promotion of colitis-associated cancer [12,52]. High MPO immunopositivity is, in turn, associated with increased incidence of colonic adenomas [53] and with microsatellite instability in adenocarcinomas [13]. Regarding enzyme association with tumor progression, high MPO expression was associated with distant metastases in head and neck cancers [54]. Moreover, MPO-including extracellular traps released by neutrophils (NET) are believed to facilitate metastasis [55].

To the best of our knowledge, there are no data concerning the dynamics of halogenated and nitrated tyrosine in the peroperative period in CRC patients. We showed distinct profiles of changes in metabolite concentration over time. While 3-BT steadily decreased and 3-CT remained constant with an increase at postoperative day three, 3-NT dropped at eight hours post-incision and then steadily increased through postoperative days one and three. As preexisting diseases may affect the body’s response to surgical trauma [56], we analyzed the potential effect of patients’ health status on modified tyrosine dynamics but found none. In turn, 3-BT dynamics were affected by the type of surgical approach, significantly dropping at eight hours post-incision solely in patients who underwent robot-assisted operation. Surgery evokes the stress response, encompassing the release of cytokines and affecting inflammation and immunity, the degree of which depends on the extent of trauma [57]. Accordingly, we and others have repeatedly shown that compared to the classic open approach, robot-assisted colorectal surgery is associated with attenuated inflammatory and beneficial immune response, which translates into favorable clinical outcomes [58,59,60,61,62,63]. No significant difference in plasma 3-NT with respect to the invasiveness of the procedure observed here is in line with previous observations concerning minilaparotomy cholecystectomy and laparoscopy cholecystectomy [64]. The 3-BT displayed distinct dynamics depending on the presence of serious postoperative complications and surgical site infections. The steady decline was observed solely in patients without these adverse effects, while patients with surgical site infections or serious complications were characterized by metabolite elevation at postoperative day one.

## 4. Materials and Methods

### 4.1. Plasma

Blood samples were collected by venipuncture, using citrate as an anticoagulant. Plasma was separated from cells by sample centrifugation at 2000× *g* for 15 min at 10 °C, aliquoted, and stored at −80 °C until investigation. Blood from CRC patients was collected preoperatively, at 8 h post-incision, and at postoperative day one and three.

### 4.2. Chemicals

3-Nitro-l-tyrosine (3-NT), 3-Chloro-l-tyrosine (3-CT), and 3-Bromo-l-tyrosine (3-BT) were procured from Sigma-Aldrich (Poznan, Poland) and Toronto Research Chemicals (Toronto, ON, Canada), respectively. Isotope-labeled 3-Nitro-l-tyrosine (RING-13C6, 99%; 3-NT-13C6), 3-Chloro-l-tyrosine (RING-13C6, 99%; 3-CT-13C6), and 3-Bromo-l-tyrosine (RING-13C6, 99%; 3-BT-13C6) were obtained from Cambridge Isotope Laboratories (Tewksbury, MA, USA). Methanol, acetonitrile, acetone, water, formic acid (FA), and trifluoroacetic acid (TFA) were acquired from Merck Millipore (Warsaw, Poland), and Leucine–enkephalin was obtained from Waters (Warsaw, Poland).

### 4.3. Calibration Standards and Plasma Sample Preparation

Standard solutions of 3-NT, 3-CT, and 3-BT were prepared in the following concentrations: 0.1, 0.2, 0.5, 1.0, 1.5, 2.0, 2.5, 3.0 ng/mL (calibration curves are presented in Figure 1). Plasma samples and calibration standards were prepared according the following procedure: aliquots of 100 µL were mixed with 10 µL of internal standard solution (12.5 ng/mL of 3-NT-13C6, 3-CT-13C6, and 3-BT-13C6 in methanol), 10 µL of 0.2% TFA, and vortexed (1 min, 25 °C). Samples were extracted using 200 µL of acetone at 25 °C for 10 min and centrifuged at 12,500 RPM for 5 min at 4 °C. The supernatant was transferred to 2 mL Eppendorf tubes and dried in a vacuum evaporator at 35 °C for 180 min. The resulting precipitate was re-dissolved in 30 µL of 0.1% FA in water, transferred to the insert, and analyzed by LC-MS/MS.

### 4.4. LC-MS/MS Analysis

LC-MS/MS was carried out using Thermo Scientific Accela UPLC system (Thermo Scientific, Waltham, MA, USA) equipped with a triple quadrupole mass analyzer (TSQ Quantum Access MAX Triple Quadrupole MS, Thermo Scientific, Waltham, MA, USA). Analytical chromatography was conducted in linear gradient mode with total flow rate of 350 µL·min^−1^ and total run time of 8.5 min on Kinetex PFP column (100 × 2.1 mm, 1.70 µm) from Phenomenex (Torrance, CA, USA). Moreover, 0.1% formic acid (FA) in water and 0.1% FA in methanol were used as mobile phase A and B, respectively. Sample injection volume was 20 µL. The applied gradient was as follows: 10% B for 0–0.7 min, 10–40% B for 0.7–5 min, 40–95% B for 5–5.5 min, 95% B for 5.5–6.5 min, and 95–10% B for 6.5–6.51 min.

The MS was equipped with an electrospray (ESI) ion source. Mass spectra were acquired in positive ionization mode. The MS parameters were as follows: the sprayer voltage, vaporizer temperature, and the capillary temperature were set at 3 kV, 265 °C, and 355 °C, respectively. Nitrogen was used as the sheath and auxiliary gas: 25 and 20 (arbitrary units), respectively. Quantitative analysis was carried out in a selected reaction monitoring (SRM) mode using the following SRM transitions: m/z 215.91 > 169.88 (CE 15 V), m/z 221.86 > 175.69 (CE 15 V); m/z 226.85 > 180.59 (CE 15 V), m/z 232.83 > 186.78 (CE 15 V); m/z 259.63 > 213.42 (CE 15 V), m/z 265.61 > 219.73 (CE 15 V) for 3-CT, 3-CT-13C6; 3-NT, 3-NT-3-NT-13C6; 3-BT, 3-BT-13C6, respectively (for ion chromatogram, please see Figure 2).

### 4.5. Characteristics of Study Population

The study group consisted of 75 patients with CRC and 43 healthy controls. Individuals with colorectal cancer were consecutive group patients undergoing surgical resection of histologically confirmed adenocarcinomas of the colon or rectum in the Department of Surgical Oncology, Regional Hospital in Wroclaw, between years 2013 and 2015. Healthy controls consisted of blood donors, whose blood samples were kindly provided by the Regional Center of Blood Donation and Therapeutics in Wroclaw, Poland. Patients with CRC preoperatively underwent a standard diagnostic workup involving colonoscopy, abdominal and pelvic computed tomography, and pelvic magnetic resonance imaging in the case of rectal cancer.

Patients with age < 18 years, ASA score > 3, emergency surgery, gross metastatic disease, cancers not amenable to curative resection, tumors requiring en bloc multi-visceral resection, coexisting malignancies, severe cardiovascular or respiratory disease, diabetes mellitus, severe mental disorders, and immunological diseases requiring systemic administration of corticosteroids were excluded.

The neoplastic disease was classified in accordance with the TNM Staging System proposed by the Union for International Cancer Control (UICC). The postoperative complications were classified with Clavien–Dindo classification [65]. Patients were operated on using traditional open approach or robot-assisted approach using the Da Vinci Si system (Intuitive Surgical, Sunnyvale, CA, USA). The choice of surgical technique was undertaken by the patient after thorough discussion with an operating surgeon. Robot-assisted procedures were performed by two colorectal surgeons with credentials in robotic surgery.

Preoperatively, all patients received mechanical bowel preparation, low molecular weight-heparin prophylaxis, and perioperative antibiotic. Parenteral opioids were used to control postoperative pain. Surgical drains were used routinely and removed on the 1st or 2nd postoperative day. Surgical site infections (SSI) were defined using Centers for Disease Control and Prevention criteria [66]. The SSIs were documented prospectively by surgical nurse and/or trained infection control personnel via telephone survey within 30 days after the surgery.

Blood was sampled at baseline (prior surgery) and at 8 h post-incision and at the first (24 h) and third (72 h) postoperative day. Samples from all four time points were available for 58 patients. Detailed characteristics of study population in an analysis at baseline and during follow-up are given in Table 4.

For the purpose of correlation analysis, data on immune and inflammatory mediators were retrieved from our database [35,36,37].

### 4.6. Ethical Approval

Study protocol was approved by the Medical Ethics Committees of Regional Specialist Hospital (#KB/nr 1/rok 2012 from 26th of June, 2012).

### 4.7. Statistical Analysis

Data normality and homogeneity of variances were tested using Kolmogorov–Smirnov and Levene tests, respectively. Data are presented as means ± SD or 95% CI or medians with 95% CI and analyzed using *t*-test for independent samples, with Welch correction if appropriate or Mann–Whitney *U* test in case of two-group comparisons or one way analysis of variance in case of multigroup comparisons. Friedman test was applied to study time profile of metabolite concentrations over time. Correlation analysis was conducted using Pearson correlation or Spearman rank correlation, depending on data character and distribution. Linear least squares regression with coefficient of determination (R^2^) indicative of the goodness of fit, Snedecor F test, and *t*-test for independent samples were used to validate LC-MS/MS method of metabolite quantification. The *p* values ≤ 0.05 were considered statistically significant. All calculated probabilities were two-tailed. The analyses were conducted using MedCalc Statistical Software version 19.5.3 (MedCalc Software Ltd., Ostend, Belgium; https://www.medcalc.org; 2020).

## 5. Conclusions

LC-MS/MS analysis of circulating nitrated and halogenated tyrosine in the selected reaction monitoring (SRM) mode using isotope-labeled analogues allows for quantitation without any or with minimal interference from tyrosine or other structurally similar metabolites. Our approach based on liquid–liquid extraction of circulating 3-NT, 3-CT, and 3-BT by acetone from plasma samples is, to the best of our knowledge, the first successful method of their simultaneous quantification using the LC-MS technique. The developed method is relatively simple and fast and characterized by satisfactory accuracy and precision and by a calibration range appropriate for the analysis of human plasma.

## Figures and Tables

**Table 1:** Results of method validation.

Compound	Sensitivity		Linearity		Precision and Accuracy						
						Within-Run (*n* = 5)			Between-Run (*n* = 10)		
	LOD (ng/mL)	LQQ (ng/mL)	R^2^	Slope tcal > tcri	Expected Conc. (ng/mL)	Measured Conc. (ng/mL)	Precision (CV%)	Accuracy (%)	Measured conc. (ng/mL)	Precision (CV%)	Accuracy (%)
3-NT	0.030	0.100	0.997	168.18 > 1.94	0.275 1.5 2.5	0.261 ± 0.006 1.52 ± 0.07 2.54 ± 0.18	9.04 3.11 2.75	94.9 101.13 101.44	0.264 ± 0.005 1.49 ± 0.07 2.52 ± 0.15	7.26 3.21 2.35	96.0 99.63 100.99
3-CT	0.026	0.096	0.996	175.39 > 1.94	0.275 1.5 2.5	0.279 ± 0.002 1.51 ± 0.11 2.61 ± 0.29	2.49 4.81 4.18	101.5 100.45 104.50	0.279 ± 0.002 1.52 ± 0.09 2.57 ± 0.24	2.51 4.05 3.59	101.5 101.63 102.84
3-BT	0.030	0.098	0.996	170.82 > 1.94	0.268 1.5 2.5	0.239 ± 0.001 1.57 ± 0.09 2.52 ± 0.19	1.52 3.61 2.97	97.5 104.73 100.77	0.270 ± 0.002 1.54 ± 0.08 2.53 ± 0.15	2.10 3.21 2.30	98.3 102.88 101.21

3-NT, 3-Nitro-l-tyrosine; 3-CT, 3-Chloro-l-tyrosine; 3-BT, 3-Bromo-l-tyrosine; LOD, limit of detection; LOQ, limit of quantification; CV, coefficient of variation; R2, regression coefficient; conc., concentration.

**Table 2:** Mean recovery rates.

Compound	Plasma Measured Concentrations (ng/mL), Mean ± SD	Response of Working Standard Solution Diluted in Water	Added Concentration (ng/mL)	Spiked Plasma Measured Concentration (ng/mL), Mean ± SD	Recovery (%) (*n* = 10)
3-NT	0.53 ± 0.08	0.097 0.209 0.429	0.30 0.66 1.36	0.87 ± 0.10 1.25 ± 0.069 2.11 ± 0.31	109.43 110.17 116.11
3-CT	1.09 ± 0.21	0.185 0.667 1.085	0.40 0.64 1.33	1.51 ± 0.31 1.66 ± 0.14 2.38 ± 0.28	106.03 90.12 96.85
3-BT	0.43 ± 0.08	0.362 0.625 1.337	0.36 0.62 1.34	0.79 ± 0.04 1.14 ± 0.08 1.76 ± 0.13	100.22 113.56 99.69

3-NT, 3-Nitro-l-tyrosine; 3-CT, 3-Chloro-l-tyrosine; 3-BT, 3-Bromo-l-tyrosine; SD, standard deviation.

**Table 3:** Plasma concentrations of modified tyrosine in colorectal cancer (CRC) patients and healthy controls.

Parameter	Controls	CRC Patients	*p* Value
*N*	43	75	-
Age, median (95%*CI*)	65 (63–68)	66 (65–70)	0.120 ^1^
Sex (F/M), *n*	22/21	30/45	0.254 ^2^
3-NT (ng/mL), mean (95%CI)	0.45 (0.32–0.58)	3.47 (1.51–5.42)	0.003 ^3^
3-CT (ng/mL), mean (95%CI)	0.52 (0.13–0.90)	1.20 (0.65–1.76)	0.044 ^3^
3-BT (ng/mL), mean (95%CI)	0.63 (0.46–0.81)	3.12 (1.42–4.82)	0.005 ^3^

*N*, number of cases; CI, confidence interval; F/M, female-to-male ratio; 3-NT, 3-Nitro-l-tyrosine; 3-CT, 3-Chloro-l-tyrosine; 3-BT, 3-Bromo-l-tyrosine; ^1^ Mann–Whitney *U* test; ^2^ Fisher exact test; ^3^ *t*-test for independent samples with Welch correction.

**Table 4:** Characteristics of study population.

Parameter	Analysis at Baseline	Follow-Up Analysis
*N*	75	58
Sex (F/M), *n*	30/45	21/37
Age (yrs.), mean (95%*CI*)	65.3 (62.4–68.3)	66.9 (63.8–69.9)
BMI, median (95%*CI*)	26.0 (25–27.6)	25.8 (24.8–27.7)
ASAPS, 1/2/3 (*n*)	17/47/11	11/38/9
CCS, median (95%*CI*)	5 (4–5)	5 (4–5)
TNM, 0/I/II/III/IV (*n*)	5/6/28/29/5	4/4/24/21/5
T, Tis/1/2/3/4 (*n*)	5/1/9/47/13	4/1/6/35/12
N, 0/1/2 (*n*)	39/20/16	32/13/13
M, 0/1 (*n*)	70/5	53/5
G, 1/2/3/4/x (*n*)	9/49/10/1/6	8/37/9/1/3
Anatomical site, RC/LC/R (*n*)	21/21/33	19/25/14
Surgery type, open/robotic (*n*)	-	33/25
Procedure, APR/LH/LAR/RH/SR (*n*)	-	2/4/23/14/15
Surgery length (min.), mean (95%*CI*)	-	174 (156–193)
Surgical site infections, yes/no (*n*)	-	45/13
Complications, CD < 3/CD ≥ 3 (*n*)	-	54/4
LoHS (days), median (range)	-	6 (3–20)

*N*, number of observations; F, females; M, males; yrs. years; *CI*, confidence interval; BMI, body mass index; ASAPS, the American Society of Anesthesiologists Physical Status Classification System; CCS, the Charlson Comorbidity Score; TNM, tumor-node-metastasis staging system; G, histopathological grade; RC, right colon; LC, left colon; R, rectum; APR, abdominal perineal resection; LH, left hemicolectomy; LAR, low anterior resection; RH, right hemicolectomy; SR, sigmoid resection; EBL, estimated blood loss; LN, lymph nodes; CDC, the Clavien–Dindo classification of postoperative complications; LoHS, length of hospital stay.
